# Photoelectrochemical Properties of CuS-GeO_2_-TiO_2_ Composite Coating Electrode

**DOI:** 10.1371/journal.pone.0152862

**Published:** 2016-04-07

**Authors:** Xinyu Wen, Huawei Zhang

**Affiliations:** 1 School of Tourism and Geographical Sciences, Yunnan Normal University, Kunming, 650500, China; 2 College of Chemistry and Chemical Engineering, Fujian Normal University, Fuzhou, 350117, China; US Naval Reseach Laboratory, UNITED STATES

## Abstract

The ITO (indium tin oxide) conductive glass-matrix CuS-GeO_2_-TiO_2_ composite coating was generated via EPD (electrophoretic deposition) and followed by a sintering treatment at 450°C for 40 minutes. Characterizations of the CuS-GeO_2_-TiO_2_ composite coating were taken by SEM (scanning electron microscope), XRD (X-ray diffraction), EDX (energy dispersive X-ray), UV-Vis DRS (ultraviolet-visible diffuse reflection spectrum), and FT-IR (Fourier transform infrared spectroscopy). Results showed that CuS and GeO_2_ had dispersed in this CuS-GeO_2_-TiO_2_ composite coating (mass percentages for CuS and GeO_2_ were 1.23% and 2.79%, respectively). The electrochemical studies (cyclic voltammetry (CV), electrochemical impedance spectroscopy (EIS) and Tafel polarization) of this CuS-GeO_2_-TiO_2_ composite coating electrode were performed in pH = 9.51 Na_2_CO_3_-NaHCO_3_ buffer solution containing 0.50 mol/L CH_3_OH under the conditions of visible light, ultraviolet light (λ = 365 nm), and dark (without light irradiation as control), respectively. Electrochemical studies indicated that this CuS-GeO_2_-TiO_2_ composite coating electrode had better photoelectrocatalytic activity than the pure TiO_2_ electrode in the electrocatalysis of methanol under visible light.

## Introduction

Since Fujishima and Honda [1] discovered the catalytic activity of the n-type TiO_2_ semiconductor electrode in the investigation of water decomposition, TiO_2_ has attracted chemists׳ attentions in the fields of heterogeneous catalytic technology [2]. TiO_2_ has excellent electrical and optical properties, chemical stability, strong oxidation ability, and non-toxicity [3–6]. However, the wide applications of TiO_2_ in heterogeneous catalysis are limited by its ultraviolet absorption (λ < 380 nm), which is caused by the large band gap between the valence band and the conduction band of TiO_2_ (~3.2 eV). Since, ultraviolet light only accounts for 8% of the solar energy, and visible light comprises 45% of the solar energy. Several different methods, such as noble metal deposition, ion doping, composite semiconductor, dye photosensitization technique [7–11], have been developed to improve the catalytic performance of TiO_2_ under visible light [12]. Semiconductor combination, as one of the methods, is regarded as the most effective method to generate TiO_2_ hybridized heterogeneous materials with excellent photocatalytic activity under visible light [13–14].

In order to improve the photoelectrocatalytic activity of TiO_2_, the CuS-GeO_2_-TiO_2_ composite coating of CuS, GeO_2_ and TiO_2_ via EPD (electrophoretic deposition) was reported in this contribution. CuS is a typical IB-VIA n-type semiconductor material with band gap at 1.2 eV, showing a strong absorption under visible light [15]. By contrast, GeO_2_ is a dielectric semiconductor oxide with a broad band (~3.4 eV) [16], which has high catalyticactivity in ultraviolet region (λ< 350 nm). Previous researches demonstrated that the mixture of a small band gap semiconductor and TiO_2_ could significantly decrease the recombination ratio between the negative photo-generated electrons (e^-^) in the conduction band and the positive holes (h^+^) in valence band, thereby could prolong the lifetime of charge carriers and extend the optical response of TiO_2_ to visible region [17].

Due to its great advantage in the modification of the thickness and microstructures of coatings and films on different substrates [18–19], EPD has been widely used in the fabrication of composite coatings and films. In this contribution, the CuS-GeO_2_-TiO_2_ composite coating on ITO conductive glass matrix was made via EPD method, and the effect of GeO_2_ and CuS on the optical response and photoelectrocatalytic activity of TiO_2_ were explored. Experimental results showed that the CuS-GeO_2_-TiO_2_ composite coating electrode had displayed on an excellent photoelectrocatalytic activity in the electrocatalysis of methanol under the irradiation of visible light. This suggested that the CuS-GeO_2_-TiO_2_ composite coating could be used in the decomposition of small organic molecules and the related field of environmental protection, and that it also could provide people with insights on the next generation of the TiO_2_ hybridized composite coating.

## Experimental Methods

### Reagents

Commercially available chemical reagents such as (C_4_H_9_O)_4_Ti, CuS, GeO_2_, Na_2_CO_3_, NaHCO_3_ and tri-ethanolamine, ethanol (99.97% purity), methanol (analytical purity), acetone (analytical purity) and n-butanol (analytical purity), polyethylene glycol (average molecular weight, 400 g/mol), and twice distilled water was used throughout the experiment.

### Instruments

CuS and GeO_2_ nanometer powders were prepared by QM-3SPO4 planetary ball mill (Nanjing University Instrument Factory, P. R. China). Ultrasonic dispersion was conducted with a KQ-100DB numerical ultrasonic cleaner (Kunshan Ultrasonic Limited Company, P. R. China). Heat treatment was conducted with a CHOY box type resistance furnace (Shanghai Experimental Second Factory, P. R. China). Electrochemical properties were measured by a CHI660C electrochemical workstation (Shanghai Chenhua Instrument Factory, P. R. China). The EPD was determined by a DYY-6B stable voltage-current electrophoresis apparatus (Beijing Instrument Factory, P. R. China). SEM with EDX were observed by S-4800 SEM (Hitachi, Japan), operating at a voltage of 20 kV. UV-Vis DRS was recorded on a Lambda 850 UV-Vis DRS (Perkin-Elmer, USA). FT-IR spectra were recorded on an Avatar-360 FT-IR spectrophotometer (Nicolet, USA) with KBr pellets of solids. The XRD pattern was recorded by an X’Pert Pro XRD (Philips, Netherlands), with Cu (Kα = 0.15418 nm) irradiation in the scan range 2θ between 10° and 80°.

### Preparation of TiO_2_ powder

The mixture of 20 ml (C_4_H_9_O)_4_Ti and 40 ml ethanol was dispersed by magnetic stirring for 10 minutes. The mixed solution was then added by dropping into 100 ml twice distilled water under the vigorous magnetic stirring [20]. Thirty minutes later, 2 ml polyethylene glycol surfactant was added into the mixture, the mixture was stirred for 10 minutes. The TiO_2_ precursor was aged at room temperature for 24 hours, and then collected by filtration. The white precipitates were washed twice with 50 ml twice distilled water and ethanol, respectively.

The white precipitates were dried at 80°C for 2 hours in the vacuum hood and then heated to 450°C at a rate of 5°C/minute in the box type resistance furnace. They were calcined at 450°C for 2 hours, and then were cooled naturally to room temperature. Finally, they were ground into fine TiO_2_ powder and stored in the dryer.

### Preparation of CuS and GeO_2_ powders

CuS and GeO_2_ powders were obtained by grinding 2 g CuS and 2 g GeO_2_ for 48 hours.

### Fabrication of CuS-GeO_2_-TiO_2_ composite coating

CuS, GeO_2_ and TiO_2_ (CuS:GeO_2_:TiO_2_ = 1:1:8) were simultaneously added into a solution containing 20 ml n-butanol (Table A in S1 File) and 1 ml tri-ethanolamine (Figure A in S1 File). The suspension was dispersed ultrasonically for 40 minutes and aged for 48 hours at room temperature to form a stable suspension.

The ITO conductive glass substrate was cleaned ultrasonically in twice distilled water by successively adding detergent, acetone and ethanol.

In the process of EPD, two parallel ITO conductive glasses at a distance of 1.0 cm were used as anode and cathode. EPD was performed by electrophoresis apparatus at 90 V (Figure B and Table B in S1 File) for 5 minutes at room temperature. The CuS-GeO_2_-TiO_2_ composite coating was obtained on anode (Table A in S1 File). In order to enhance the bonding strength between a CuS-GeO_2_-TiO_2_ composite coating and ITO conductive glass matrix, the sintering treatment was performed at 450°C for 40 minutes, and then was cooled down to room temperature. Ultimately, a CuS-GeO_2_-TiO_2_ composite coating was obtained with a thickness of approximately 0.2 cm.

### Electrochemical measurements of CuS-GeO_2_-TiO_2_ composite coating electrode

Electrochemical measurements were performed with a three-electrode system, the saturation calomel electrode (SCE) was used as a reference electrode, the platinum electrode served as a counter electrode, and the CuS-GeO_2_-TiO_2_ composite coating electrode was used as a working electrode. The Na_2_CO_3_-NaHCO_3_ buffer solution (pH = 9.51) containing 0.50 mol/L CH_3_OH (electroactive species) was used as electrolyte. All measurements were conducted at room temperature.

## Results and Discussion

### SEM images of CuS-GeO_2_-TiO_2_ composite coating

**Fig 1:**
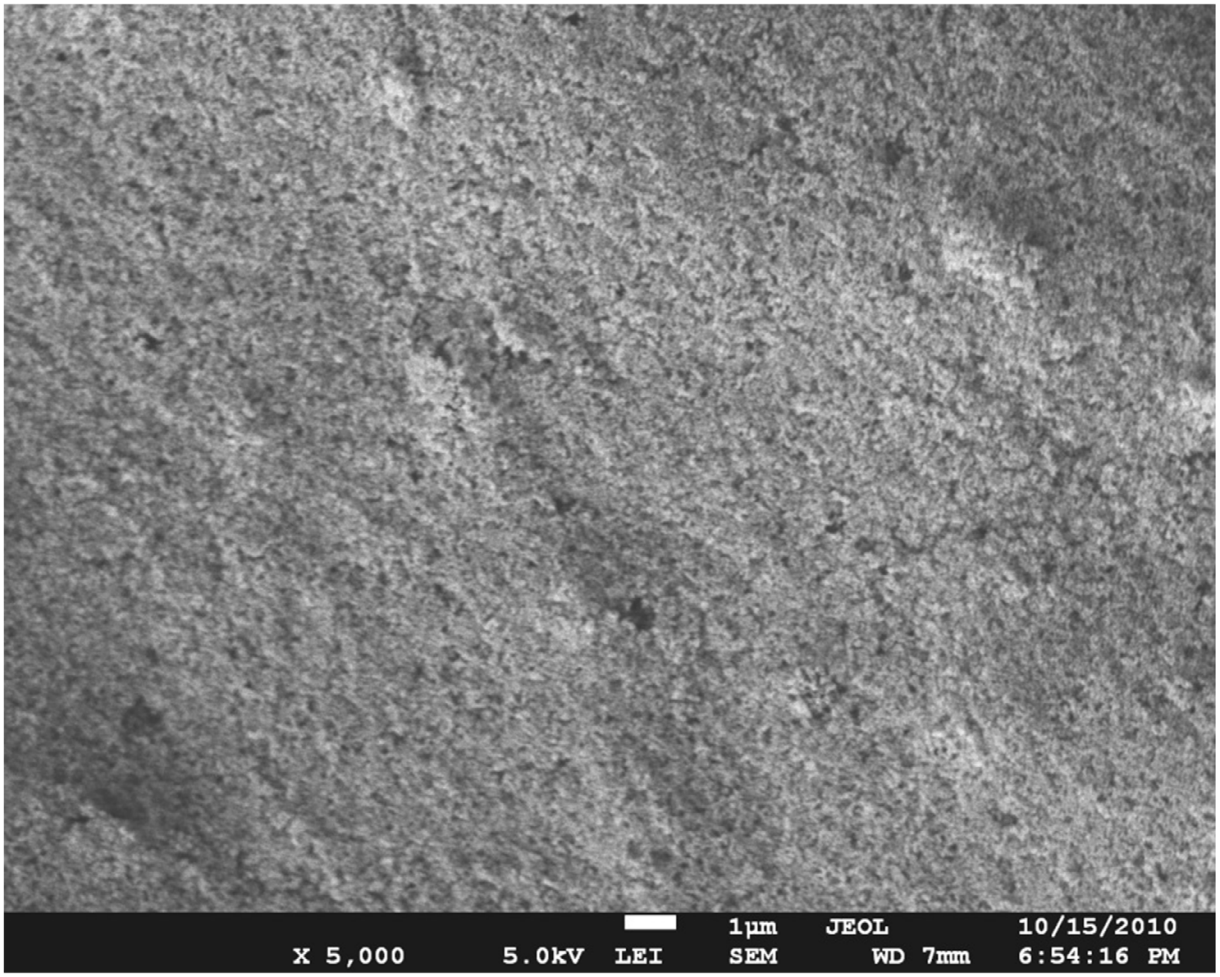
SEM morphology of CuS-GeO_2_-TiO_2_ composite coating.

SEM morphology of the CuS-GeO_2_-TiO_2_ composite coating surface was displayed in Fig 1, demonstrating that the CuS-GeO_2_-TiO_2_ composite coating exhibited a rough surface with multiple holes after the sintering treatment at 450°C. This structure can increase the contact interface between the CuS-GeO_2_-TiO_2_ composite coating and light source, which is beneficial to photochemical reactions [21]. Furthermore, it verified that TiO_2_, CuS and GeO_2_ were uniformly distributed on ITO conductive glass surface with particle sizes of approximately 0.5–1.0 μm.

### EDX analysis of CuS-GeO_2_-TiO_2_ composite coating

**Fig 2:**
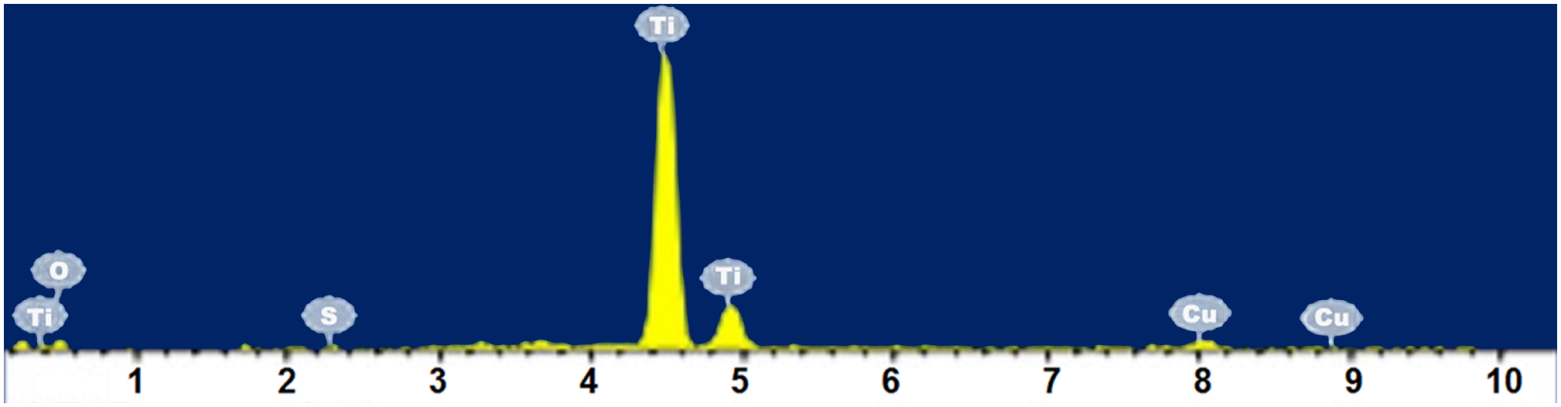
EDX spectrum of CuS-GeO_2_-TiO_2_ composite coating.

EDX analysis confirmed the presence of CuS and GeO_2_ particles in the CuS-GeO_2_-TiO_2_ composite coating (Fig 2). All elements of the CuS-GeO_2_-TiO_2_ composite coating were observed in EDX spectrum, demonstrating that the CuS-GeO_2_-TiO_2_ composite coating was comprised of CuS, GeO_2_ and TiO_2_. The mass percentages of CuS and GeO_2_ in the CuS-GeO_2_-TiO_2_ composite coating (Table 1) were 1.23% and 2.79%, respectively, indicating that CuS and GeO_2_ were successfully co-deposited with TiO_2_. These results suggested that the co-deposition of CuS, GeO_2_ and TiO_2_ on ITO conductive glass surface formed the CuS-GeO_2_-TiO_2_ composite coating.

**Table 1:** EDX data of CuS-GeO_2_-TiO_2_ composite coating.

Elements	Mass percentage(%)	Atom percentage(%)
O	17.50	39.35
Ti	74.86	56.23
Cu	5.29	3.00
S	0.41	0.46
Ge	1.94	0.96
Total	100.00	100.00

### Structure analysis of CuS-GeO_2_-TiO_2_ composite coating

**Fig 3:**
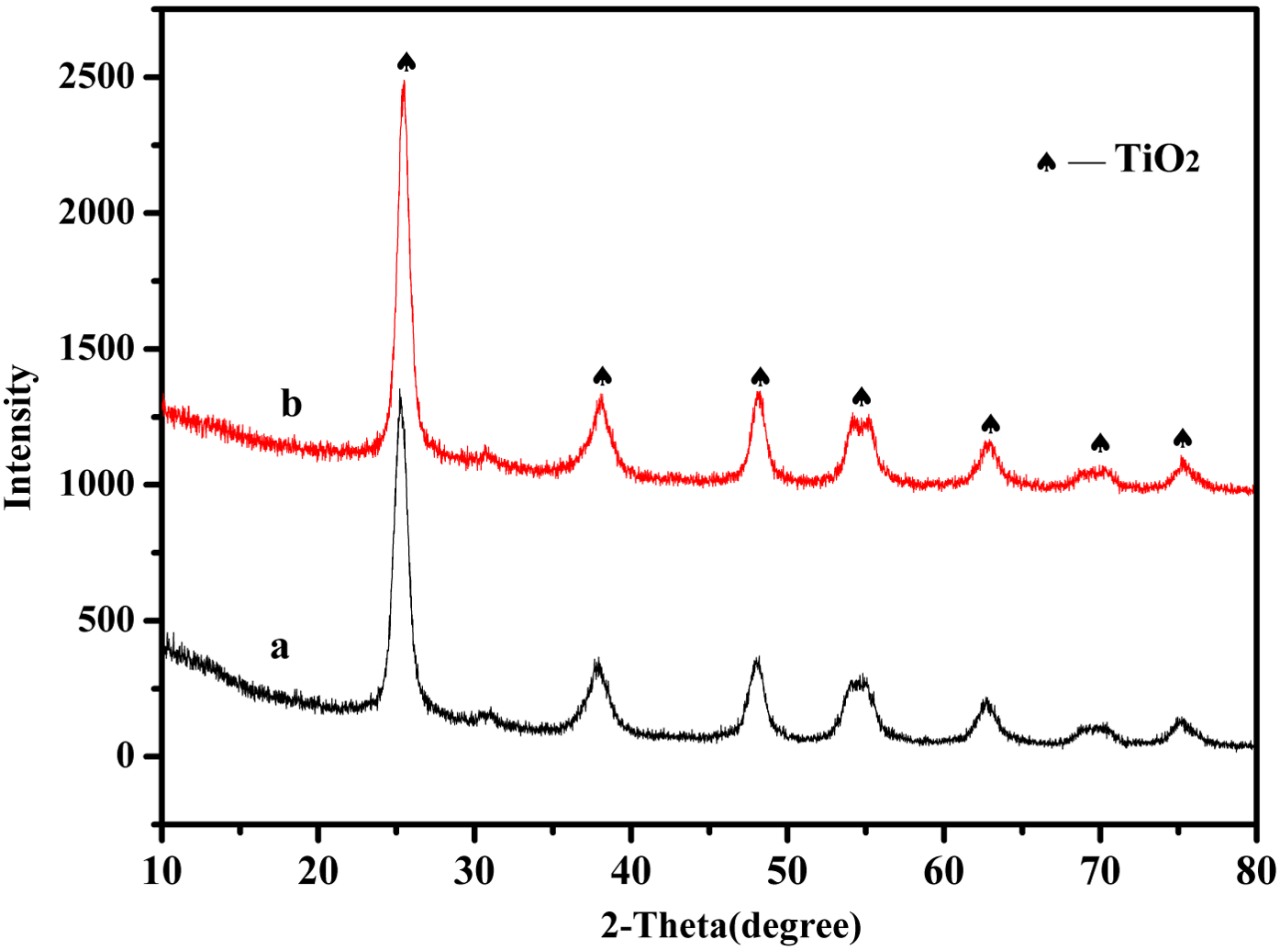
XRD patterns of CuS-GeO_2_-TiO_2_ composite coating (a) and TiO_2_ (b).

Fig 3 illustrated XRD patterns of the pure TiO_2_ (Fig 3b) and the CuS-GeO_2_-TiO_2_ composite coating (Fig 3a). Pure TiO_2_ and the CuS-GeO_2_-TiO_2_ composite coating both consisted of anatase TiO_2_ crystalline consistent with reference [22], illustrating that the crystalline phase of TiO_2_ was not altered in the CuS-GeO_2_-TiO_2_ composite coating after the sintering treatment. Due to the relatively low content of CuS and GeO_2_ compared to a large amount of TiO_2_, the significant diffraction peaks of crystalline CuS and GeO_2_ crystalline were not observed.

### FT-IR spectrum of CuS-GeO_2_-TiO_2_ composite coating

**Fig 4:**
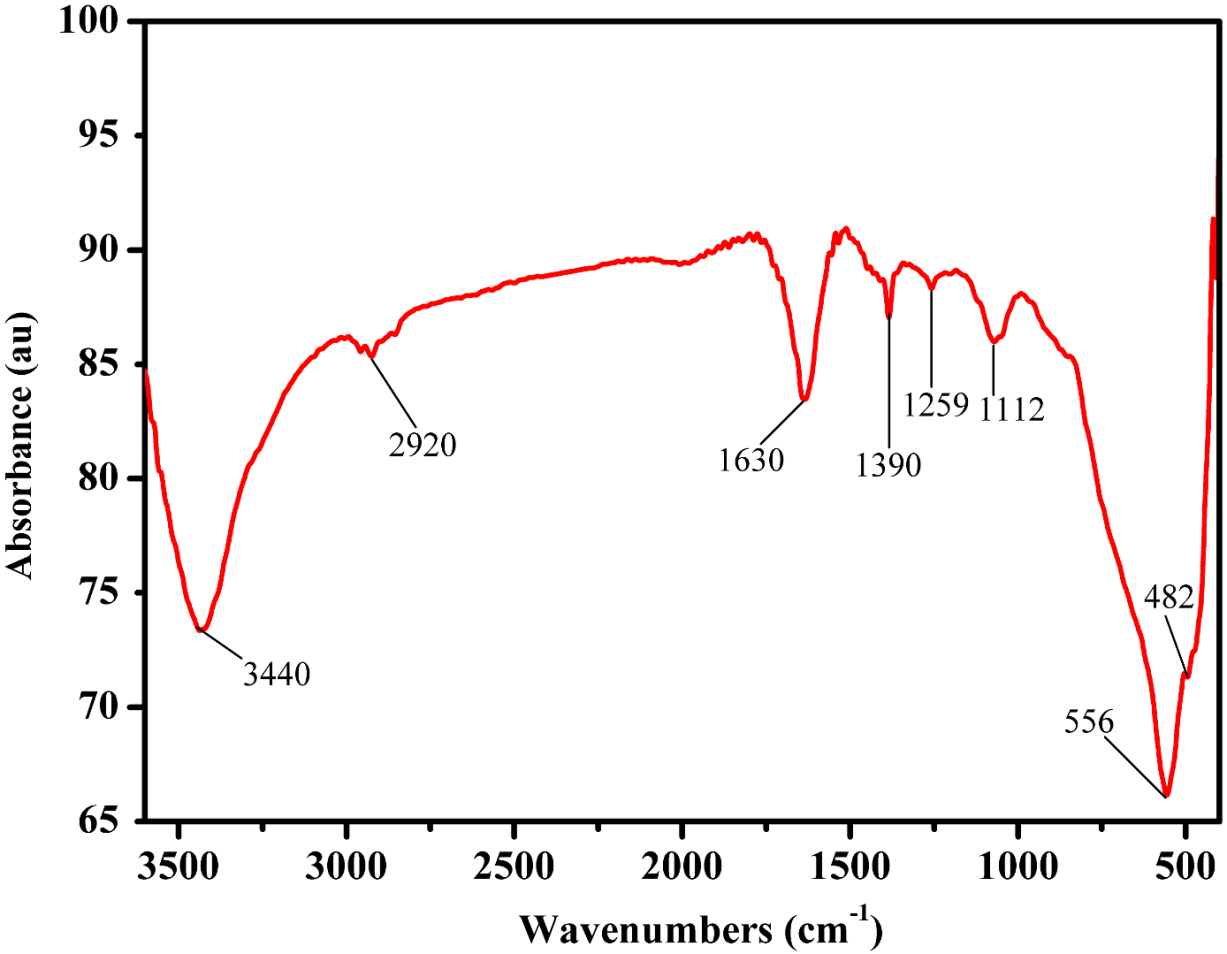
FT-IR spectrum of CuS-GeO_2_-TiO_2_ composite coating.

The FT-IR spectrum of the CuS-GeO_2_-TiO_2_ composite coating was displayed in Fig 4. In the FT-IR spectrum, the strong absorption band at 3440 cm^-1^ was the O-H stretching vibration of absorbed water attached to the surface of the CuS-GeO_2_-TiO_2_ composite coating. Another typical absorption band at 1630 cm^-1^ was assigned to H-O-H bending vibration [23]. The vibration modes of the anatase skeleton structure of Ti-O-Ti bonds were observed in the range of 500 to 900 cm^-1^, with a maximum of 556 cm^-1^ which was assigned as Ti-O-Ti characteristic absorption peak [24]. The absorption band observed at 1112 cm^-1^ was ascribed to the characteristic absorption peak of CuS [25], and the much weaker absorption band at 482 cm^-1^ was the characteristic absorption peak of GeO_2_ [26]. Considering the electron affinity of O, Ge and S elements, the weak absorption band at 1390 cm^-1^ could be assigned to S-O-Ge, suggesting that a slight conjugation effect existed among CuS, GeO_2_ and TiO_2_ [25]. Furthermore, the weaker absorption band at 1259 cm^-1^ might be related to the vibration of S-O-Ge bonds, as observed in reference [26].

### UV-Vis DRS of CuS-GeO_2_-TiO_2_ composite coating

**Fig 5:**
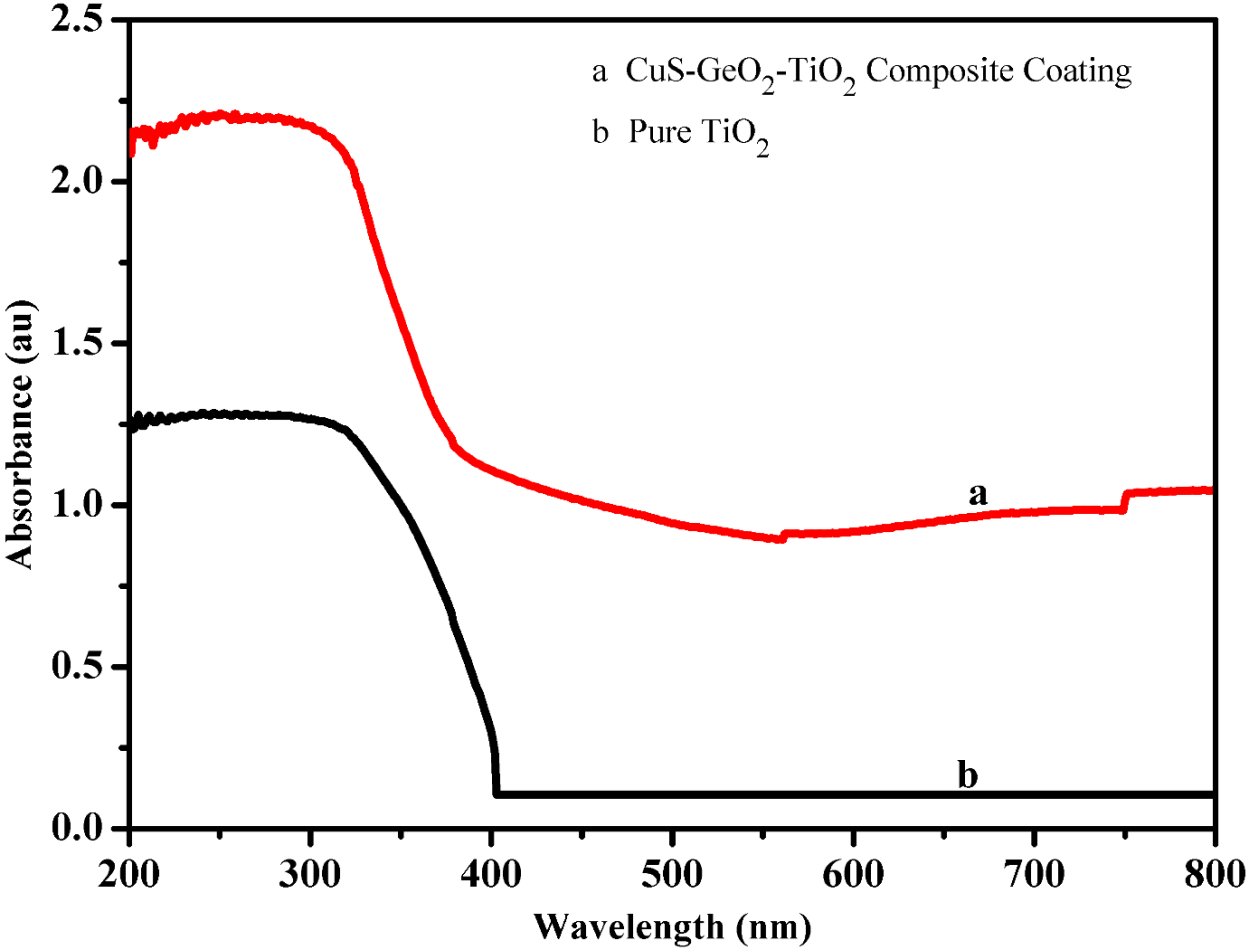
UV-Vis DRS of CuS-GeO_2_-TiO_2_ composite coating (a) and pure TiO_2_ (b).

The CuS-GeO_2_-TiO_2_ composite coating (Fig 5a) showed unique absorptions in ultraviolet and visible region compared to pure TiO_2_ (Fig 5b). The CuS-GeO_2_-TiO_2_ composite coating had the similar spectral profile in ultraviolet region as pure TiO_2_. Whereas, the presence of CuS and GeO_2_ increased the absorption intensity of the CuS-GeO_2_-TiO_2_ composite coating in visible region. In ultraviolet region, the absorption of the CuS-GeO_2_-TiO_2_ composite coating was attributed to the intrinsic band gap of pure anatase TiO_2_ (∼3.2 eV) and GeO_2_ (~3.4 eV), beginning at a wavelength shorter than 380 nm. In visible region, the absorption between 380 and 800 nm was attributed to CuS. The optical response of visible region associated with CuS dopants could be ascribed to the electron exchange among CuS, GeO_2_ and TiO_2_. Electrons first migrated from CuS conduction band to TiO_2_ conduction band, and then moved to GeO_2_ conduction band. Meanwhile, holes first transferred from GeO_2_ valence band to TiO_2_ valence band, and then moved to CuS valence band. The electrons and holes significantly prolonged the lifetime of photo-induced carriers and extended the optical response to visible region. Thus, the CuS-GeO_2_-TiO_2_ composite coating could increase absorption in visible region, and could possibly increase the photoelectrocatalytic activity under visible light [27] compared to pure TiO_2_.

### CV measurements of CuS-GeO_2_-TiO_2_ composite coating electrode

**Fig 6:**
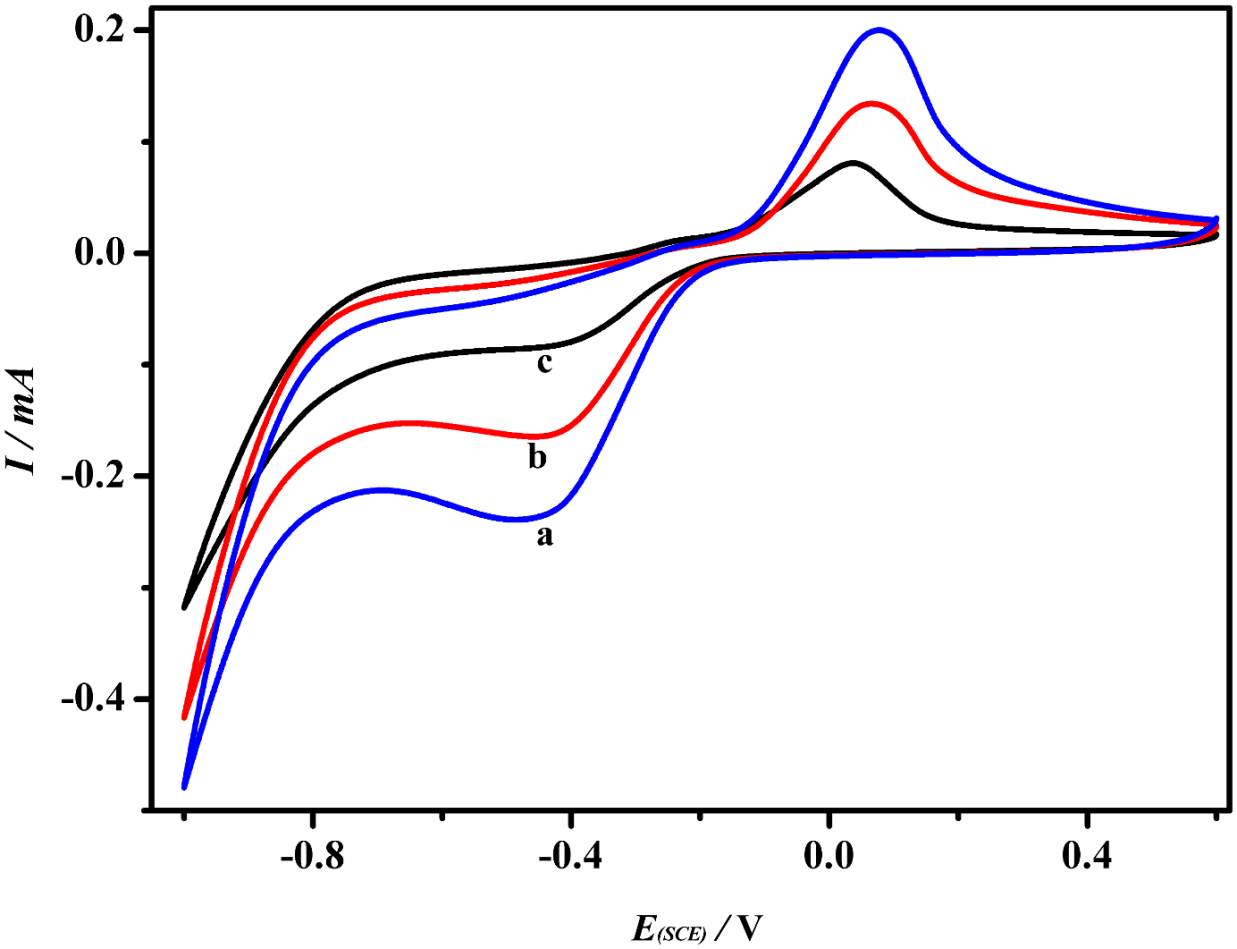
CV of CuS-GeO_2_-TiO_2_ composite coating electrode (a) Ultraviolet light, (b) Visible light, and (c) Dark.

Fig 6 presented the results of cyclic voltammetry (CV) measurements in Na_2_CO_3_-NaHCO_3_ buffer solution (pH = 9.51) with 0.50 mol/L CH_3_OH using the CuS-GeO_2_-TiO_2_ composite coating as a working electrode. The oxidation potential of methanol began at approximately 0.07 V (*vs*. SCE), while the reduction potential was at about -0.42 V (*vs*. SCE). The former perhaps corresponded to the adsorption of the carbonyl compounds (e.g. TiO_2_-CH_2_OH, TiO_2_-CHO, TiO_2_-COH, TiO_2_-CO, TiO_2_-CHOH) [28], belonging to the possible oxidation products of methanol on the CuS-GeO_2_-TiO_2_ composite coating electrode. The latter was ascribed to the reduction of the carbonyl compounds to alcohols or aldehydes compounds [28]. In addition, under visible light (Fig 6b), the potential negatively shifted about 0.03 V and the current density remarkably increased about 0.05 mA compared to dark control (Fig 6c). The excellent photoelectrocatalytic activity of the CuS-GeO_2_-TiO_2_ composite coating electrode could be explained by the efficient separation of photo-generated e^-^-h^+^ under visible light. During the electrons transferring from the valance band to the conduction band of the CuS-GeO_2_-TiO_2_ composite coating under visible light, the valance band could form positive h^+^ and the conduction band was occupied by highly active e^-^, which formed photoelectrocatalysis active center. In the electrolyte, the electroactive species methanol acted as photo-generated holes trapper to trap adsorbing carbonyl compounds adsorbed h^+^. And then, methanol accepted e^-^ and prompted the reduction of the carbonyl compounds.

### EIS analysis of CuS-GeO_2_-TiO_2_ composite coating electrode

**Fig 7:**
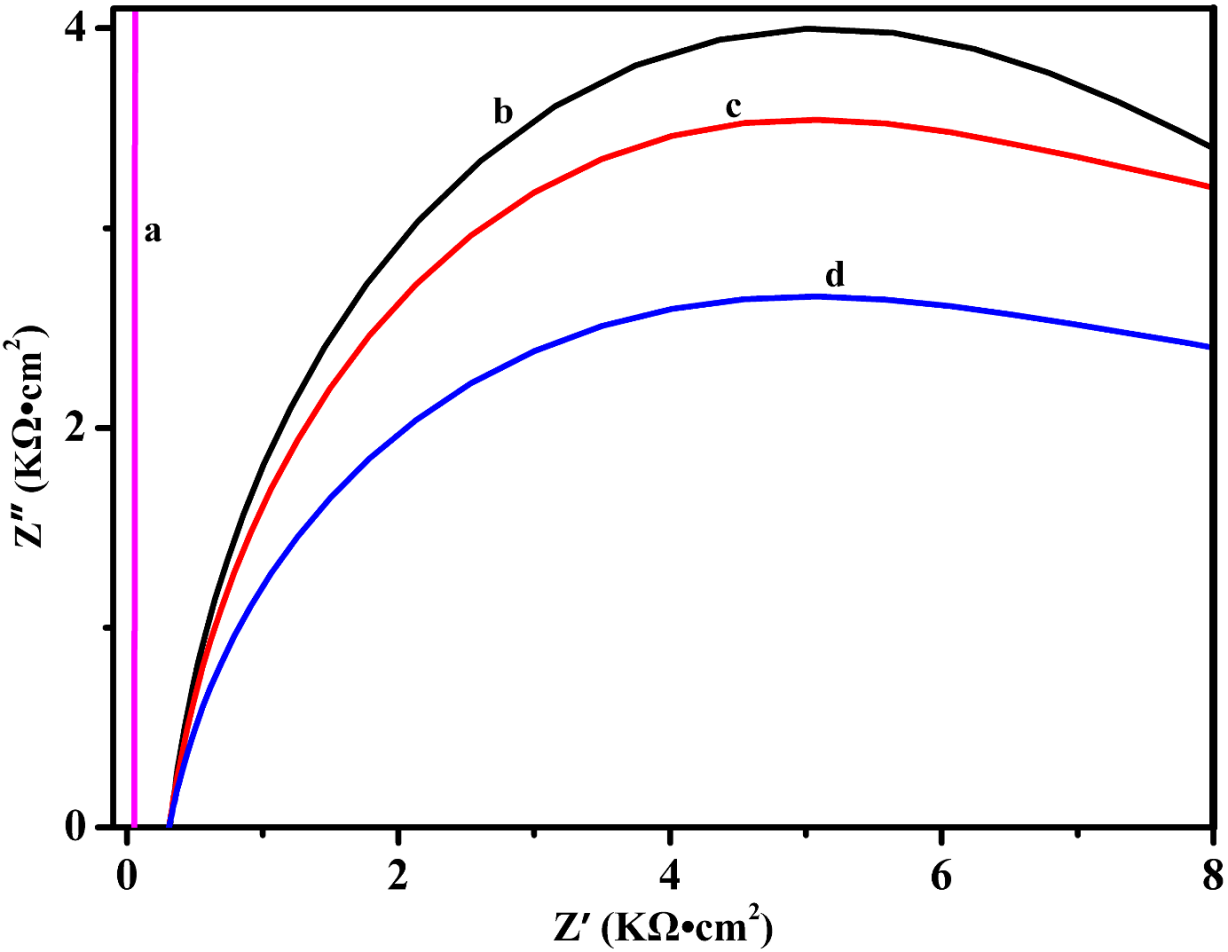
EIS of CuS-GeO_2_-TiO_2_ composite coating electrode (a) PureTiO_2_, (b) Dark, (c) Visible light, and (d) Ultraviolet light.

EIS (electrochemical impedance spectroscopy) measurement was performed to study the interaction of the CuS-GeO_2_-TiO_2_ composite coating electrode/electrolyte interface. The EIS experiments were performed in a frequency range of 0.01-10^5^ Hz. Typical Nyquist plots of EIS measurements were showed in Fig 7, which compared the imaginary component Z” to the real component Z’.

For the pure TiO_2_ electrode, the imaginary component Z” was perpendicular to the real component Z’ (Fig 7a), indicating that TiO_2_ electrode exhibited the property of a resistance in high-frequency area and the property of a capacitance in low-frequency area. The semicircle Nyquist plots of the CuS-GeO_2_-TiO_2_ composite coating electrode showed that the impedance circle radius was largest under the dark condition (Fig 7b, 7c and 7d). The largest impedance circle radius under the dark condition was caused by the charge transfer resistance, suggesting that the electrocatalysis of methanol dominated by electrochemical process rather than diffusion. The smaller radius of the impedance circle under visible light suggested that the CuS-GeO_2_-TiO_2_ composite coating electrode had a better electrocatalytic activity than in dark control. This might be attributable to the prolonged lifetime of the photo-generated charge carriers by doping semiconductor materials into TiO_2_.

**Fig 8:**
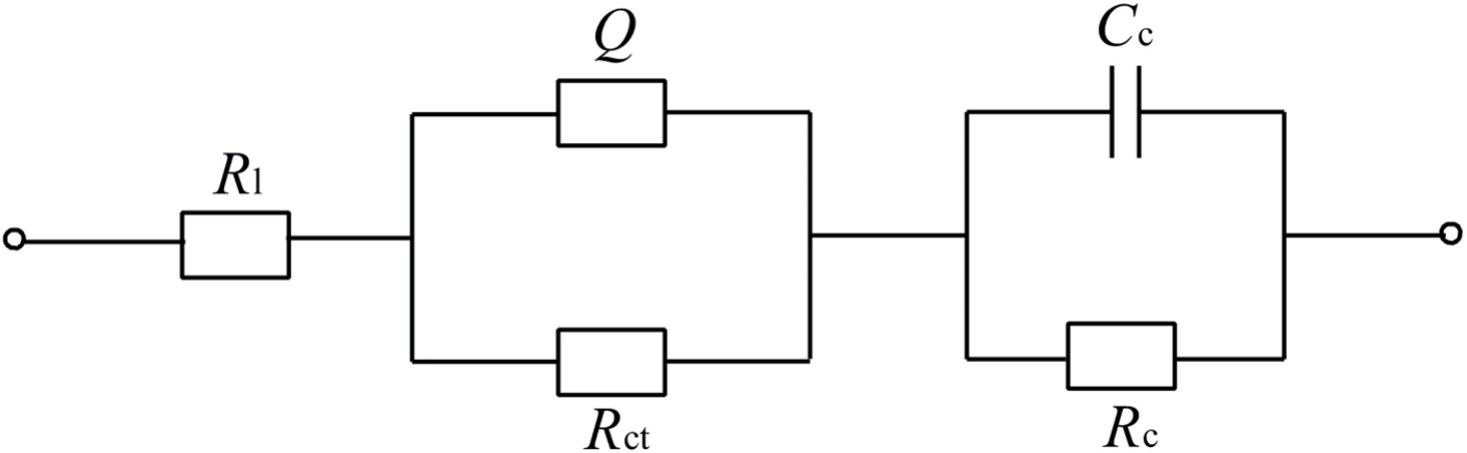
EIS equivalent circuit of CuS-GeO_2_-TiO_2_ composite coating electrode.

The equivalent circuit modes applied to fit the experimental EIS date of the CuS-GeO_2_-TiO_2_ composite coating electrode was shown in Fig 8. In this case, *R*_*l*_ referred to the electrolyte resistance, *R*_*ct*_ referred to the charge transfer resistance, *Q* referred to the constant phase component, which was the combination of properties related to the surface and electroactive species (methanol), *C*_*c*_ referred to the double-layer capacitance of the CuS-GeO_2_-TiO_2_ composite coating electrode; and *R*_*c*_ referred to the resistance of the CuS-GeO_2_-TiO_2_ composite coating electrode. The fitting results of all parameters of the equivalent circuit were listed in Table 2.

**Table 2:** The fitting results of all parameters of the equivalent circuit model for EIS data.

Parameters	under the dark	under the visible light	under the ultraviolet light
*R*_*l*_(KΩ·cm^2^)	10.23(3.485)	9.328(4.284)	5.752(3.553)
*Q*(S·sec^n·cm^-2^)	1.143×10^-2^(3.747)	1.691×10^-4^(3.666)	3.144×10^-5^(4.424)
*R*_*ct*_(KΩ·cm^2^)	1.056(9.186)	9.402(8.16)	0.3219(1.954)
*C*_*c*_(F·cm^-2^)	1.847×10^-5^(3.914)	1.662×10^-5^(3.941)	1.607×10^-4^(9.841)
*R*_*c*_(KΩ·cm^2^)	0.4885(3.543)	0.5089(3.579)	2.154(7.135)

The data in parentheses are the simulating error

### Tafel polarization curves of CuS-GeO_2_-TiO_2_ composite coating electrode

**Fig 9:**
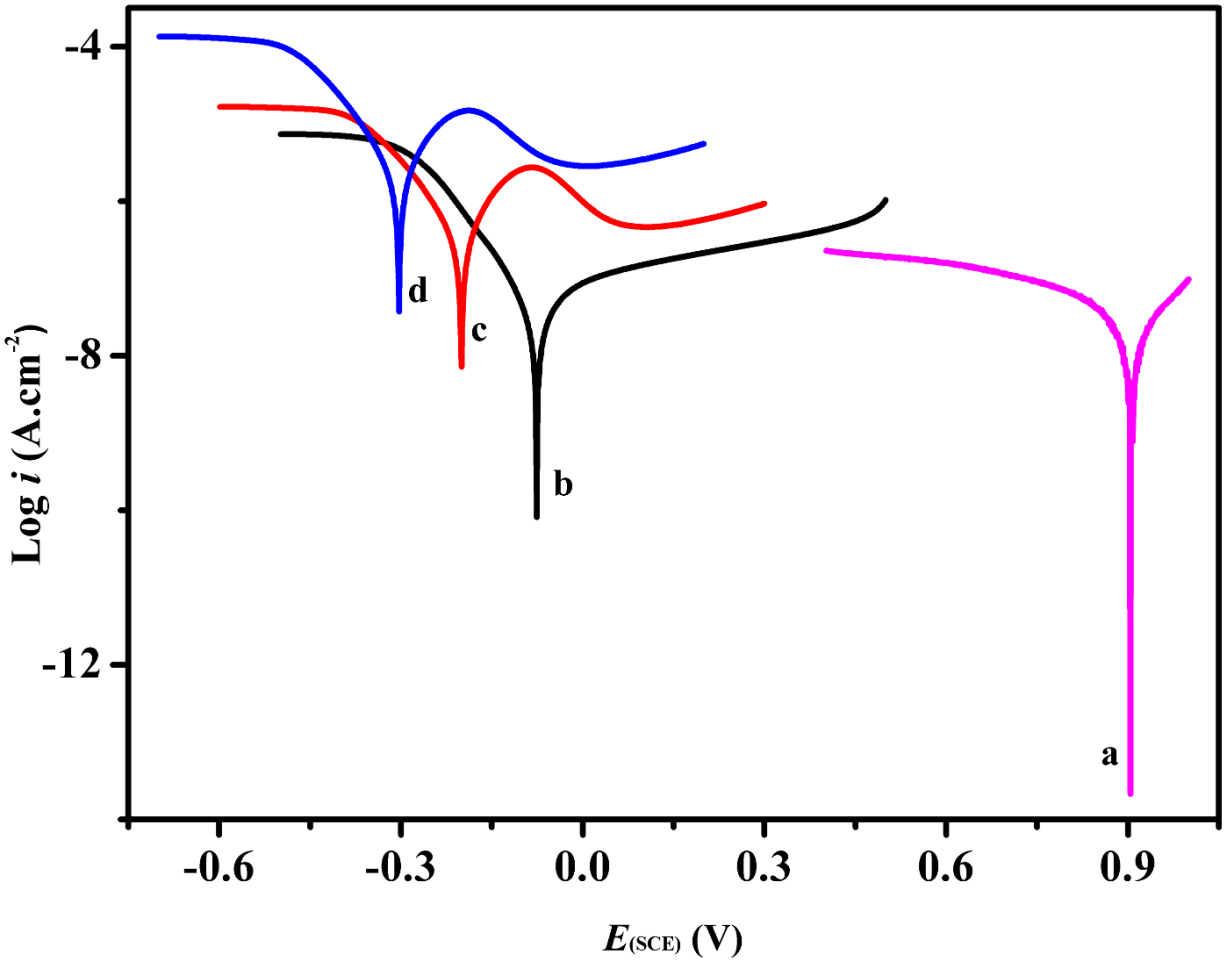
Tafel polarization curves of CuS-GeO_2_-TiO_2_ composite coating electrode. (a) Pure TiO_2_, (b) Dark, (c) Visible light, and (d) Ultraviolet light.

Fig 9 showed the Tafel polarization curves of the CuS-GeO_2_-TiO_2_ composite coating electrode in Na_2_CO_3_-NaHCO_3_ buffer solution (pH = 9.51) with 0.50 mol/L CH_3_OH in dark (Fig 9b), under visible light (Fig 9c) and under ultraviolet light (Fig 9d). Under visible light (Fig 9c), the polarization current density of the CuS-GeO_2_-TiO_2_ composite coating electrode increased to 10^-6.9^ A/cm^2^, and the polarization potential negatively shifted by about 1.1 V compared to the pure TiO_2_ electrode (Fig 9a), which demonstrated that the electrochemical activity of the CuS-GeO_2_-TiO_2_ composite coating electrode was much higher than that of the pure TiO_2_ electrode (Fig 9a). This may be because of the electrochemical activity enhancement of the CuS-GeO_2_-TiO_2_ composite coating electrode under visible light [29]. This also indicated that the CuS-GeO_2_-TiO_2_ composite coating electrode greatly improved the absorptive capacity of visible light. In addition, at the polarization current density, the potential was -0.08 V (vs. SCE) in dark (Fig 9b), while it was -0.20 V (vs.SCE) under visible light (Fig 9c). Therefore, the visible light could optimally promote the photoelectrocatalysis of methanol on the CuS-GeO_2_-TiO_2_ composite coating electrode.

## Conclusions

1. The CuS-GeO_2_-TiO_2_ composite coating on ITO conductive glass surface was successfully generated via EPD. CuS and GeO_2_ were evenly dispersed in the CuS-GeO_2_-TiO_2_ composite coating with approximate particle size at 0.5–1.0 μm.
2. The optical response of the CuS-GeO_2_-TiO_2_ composite coating significantly extended to the visible region.
3. The CuS-GeO_2_-TiO_2_ composite coating electrode displayed excellent photocatalytic activity under visible light, which greatly improved the photoelectrocatalysis of methanol and performed a greatly anodic and cathodic photo-generated current.
4. The CuS-GeO_2_-TiO_2_ composite coating electrode might be used as a photoelectrocatalytic material in the decomposition of small organic molecule, such as direct methanol fuel cell (DMFC), and the related field of environmental protection with high efficient sunlight utilization.

## Supporting Information

S1 File This File includes the choice of the dispersed medium, charged particles analysis, ITO conductive glass, and selection of the electric field intensity in the EPD (electrophoretic deposition) process. Figure A. Relationships of electric conductivity *vs*. aging time in CuS-GeO_2_-TiO_2_ suspensions (a: adding TEA b: unadding TEA). Figure B. Plot of the mass of CuS-GeO_2_-TiO_2_ composite coating *vs*. electric field intensity in EPD (electrophoretic deposition). Table A. The influence of solvent on morphology of TiO_2_ sedimentary sequences and electrode. Table B. The effect of electric field intensity on morphology of CuS-GeO_2_-TiO_2_ composite coating.
(DOCX)
