# Supplementary material for: Photoelectrochemical Properties of CuS-GeO2-TiO2 Composite Coating Electrode
Source: PLoS One. 2016 Apr 7;11(4):e0152862. doi: 10.1371/journal.pone.0152862 (PMC4824510; doi:10.1371/journal.pone.0152862)
Supplement: S1 File — Figure A. Relationships of electric conductivity vs. aging time in CuS-GeO2-TiO2 suspensions (a: adding TEA b: unadding TEA). Figure B. Plot of the mass of CuS-GeO2-TiO2 composite coating vs. electric field intensity in EPD (electrophoretic deposition). Table A. The influence of solvent on morphology of TiO2 sedimentary sequences and electrode. Table B. The effect of electric field intensity on morphology of CuS-GeO2-TiO2 composite coating. (DOCX) [file pone.0152862.s001.docx]

**Support Information for**

**Photoelectrochemical Properties of CuS-GeO_2_-TiO_2_ Composite Coating Electrode**

Xinyu Wen^1,2^; Huawei Zhang^1^*

*Corresponding author, hwzhang@ynnu.edu.cn

1. The choice of the dispersed medium

Different organic solvents as dispersants made TiO_2_ particles to have different charges, so that TiO_2_ coating was deposited on the different electrodes surface. At 90 V/cm [electric field intensity](http://dict.cnki.net/dict_result.aspx?searchword=%e7%94%b5%e5%9c%ba%e5%bc%ba%e5%ba%a6&tjType=sentence&style=&t=electric+field+intensity), TiO_2_ concentration was 5.0 mg/ml, the distance between two electrodes was 0.5 cm, the time of EPD (electrophoretic deposition), and the effect of the different solvents on morphology of the TiO_2_ deposition coating were listed in Table A.

Table A. The influence of solvent on morphology of TiO_2_ sedimentary sequences and electrode

| Solvent | Depositing Electrode | Morphology of TiO_2_ Sedimentary Sequence |
| --- | --- | --- |
| Methanol | Anode | The deposition coating was very thin, the reduction reaction occurred on the cathode of the ITO conductive glass surface with black substance generated. |
| Ethanol | Anode | The deposition coating was very thin and uneven. End effect was serious, the reduction reaction occurred on the cathode of the ITO surface. |
| n-propanol | Anode | The deposition coating was very thin, loose and uneven. |
| Isopropanol | Cathode | The deposition coating was very loose and uneven. |
| n-butanol | Cathode | The deposition coating was uniform and dense. |
| Pentanol | Cathode | The deposition coating was very thin. |
| Acetone | Anode | The deposition coating was very thin and uneven. End effect was serious. |
| Ethyl acetate | Anode | The deposition coating was very thin and uneven. End effect was serious. |
|  | | |

Methanol, ethanol and n-propanol are protonic solvents, when they are respectively used as dispersion medium, TiO_2_ suspended particles are deposited on the anode of the ITO conductive glass surface, illustrating that TiO_2_ have a strong interaction with the three kinds of dispersion media. Moreover, the effect is realized by the oxygen atom in the H-O of the alcohol molecules. Since oxygen atom contains lone pair electron having higher electronic gativity, the solvation between TiO_2_ particles and H-O oxygen atom occurs in the suspension which make TiO_2_ to have negative charge. Compared to n-propanol, TiO_2_ suspension particles are deposited on the cathode of the ITO surface, mainly because there is large steric hindrance in isopropanol molecule, which is beneficial to form strong solvation between hydrogen atom of O-H and TiO_2_ particles to make TiO_2_ particles with positive charge.

Acetone and ethyl acetate are non-protonic solvents, when they are respectively used as the dispersion medium, the solvation is realized by oxygen atom of C=O, so the coating is deposited on the anode of ITO conductive glass surface. Protonic solvents are used as dispersion medium, with the number of carbon atoms increase, TiO_2_ suspension particles are deposited on the anode or the cathode, and isopropanol is turning point. Alcohol as polar solvent can significantly reduce the surface energy of the oxides, so that the coating is deposited faster and firmly on ITO conductive glass electrode. During EPD process, n-butanol is the best dispersion medium as listed in Table B.

Table B. The effect of electric field intensity on morphology of CuS-GeO_2_-TiO_2_ composite coating

| Electric Field Intensity (V/cm) | Morphology of Composite Coating |
| --- | --- |
| 20 | ITO conductive glass surface did not contain any substance. |
| 40 | ITO conductive glass surface did not contain any substance. |
| 60 | The deposition coating was very thin and uneven. End effect was serious. |
| 80 | The deposition coating was uniform and dense. |
| 100 | The deposition coating was uniform and dense. |
| 110 | The deposition coating was very thick, loose and rough surface. SnO_2_ in the ITO conductive glass was reduced to Sn and black substance was generated. |

2. Charged particles analysis

Dried TiO_2_ were grinded into nanometer by agate mortar. 0.100 g TiO_2_ and a certain amount of CuS and GeO_2_ were added into 20 ml n-butanol, the suspension was added 1.0 ml and 0 ml TEA (tri-ethanolamine), respectively. The mixed solution was then subjected to ultrasonic dispersion. Electric conductivity of the adding and unadding TEA suspension changed with aging time as shown in Figure A**.**

ITO conductive glass was severed as anode and cathode, respectively. The deposition coating was found to form on the cathode of the ITO conductive glass, illustrating that TiO_2_, CuS and GeO_2_ were positively charged in the suspension. All the three charged were due to n-butanol dissociation and/or proton exchange. The electric conductivity of the adding TEA suspension of CuS-GeO_2_-TiO_2_ increased nearly three-fold, which is beneficial to EPD.





Figure A. Relationships of electric conductivity *vs*. aging time in CuS-GeO_2_-TiO_2_ suspensions

(a: adding TEA b: unadding TEA)

3. ITO conductive glass

ITO (Indium-Tin-Oxide) conductive glass is a transparent conductive oxide film. Generally, semiconductor oxides, such as SnO_2_ and In_2_O_3_, are coated on the ITO surface [1]. The electrical conductivity of the ITO conductive glass is almost close to metal, its value is 10^-4^S/cm. Due to the low electrical conductivity of ITO conductive glass, it has a very low reflectivity (<10%) in the visible and ultraviolet region. In addition, ITO conductive glass has some advantages, such as resistance to chemical corrosion, easily etching and semiconductor characteristic [2]. In this experiment, ITO conductive glass played the role of electronic transmission and collection, and provided the reaction field for the oxidation-reduction reaction.

4. Selection of the electric field intensity in the EPD process

Their concentrations including TiO_2_, CuS and GeO_2_ were 5.00 mg/ml, 0.50 mg/ml and 0.50 mg/ml, respectively. Deposition time was 5 minutes, the distance between two electrodes was 1.0 cm, two ITO conductive glass was used as anode and cathode, respectively. The surface morphology and the deposition amount of CuS-GeO_2_-TiO_2_ composite coating prepared were showed in Table A and Figure B.

When the electric field was located between 80 V/m and 100 V/m, the mass of CuS-GeO_2_-TiO_2_ composite coating at the unit time was no significant increase, so the electric field must be controlled at 80~100 V/m to obtain uniform and compact CuS-GeO_2_-TiO_2_ composite coating.





Figure B. Plot of the mass of CuS-GeO_2_-TiO_2_ composite coating *vs*. electric field intensity in EPD

**References**

1. Ma Y, Zhang F H, Mu Q. Properties, preparation and applications of the transparent and conductive glass with ITO film. J. Shaanxi University of Science & Technology. 2003; 21(1): 106-109.

2. Masato S, Masatoshi H. Electric properties of ITO films prepared by tin ion implantation in Ln_2_O_3_ films. Thin Solid Films. 1998; 317: 157-160
